# Ammonia Synthesis Over an Iron Catalyst with an Inverse Structure

**DOI:** 10.1002/advs.202410313

**Published:** 2025-01-23

**Authors:** Masashi Hattori, Kento Miyashita, Yuki Nagasawa, Ryo Suzuki, Michikazu Hara

**Affiliations:** ^1^ Materials and Structures Laboratory Institute of Integrated Research Institute of Science Tokyo 4259 Nagatsuta, Midori‐ku Yokohama 226‐8503 Japan; ^2^ F.C.C. CO., LTD. 7000‐36 Nakagawa, Hosoe‐cho, Hamana‐ku Hamamatsu Shizuoka 431‐1304 Japan

**Keywords:** ammonia synthesis, electron‐donating materials, haber‐bosch process, heterogeneous catalyst, iron catalyst

## Abstract

Achieving a substantial increase in the ammonia productivity of the Haber‐Bosch (HB) process at low temperatures has been a significant challenge for over 100 years. However, the iron catalyst designed over 100 years ago remains at the forefront of this process because it is difficult to exceed the industrial iron catalyst in terms of the ammonia synthesis rate/catalyst volume that determines ammonia productivity in a reactor. Here, a new catalyst with an inverse structure of a supported metal catalyst that consists of metallic iron particles loaded with an aluminum hydride species is reported. The iron catalyst is readily prepared from an α‐Fe_2_O_3_ precursor and ammonia could be synthesized at more than twice the ammonia synthesis rate/catalyst volume of the conventional industrial iron catalyst, even though the specific surface area of the former is only half that of the latter. In addition, ammonia synthesis over the catalyst is observed with a small apparent activation energy at 50 °C. Mechanistic studies suggested that an increase in the active sites with strong electron‐donating capability on the iron catalyst significantly increased the ammonia synthesis rate/catalyst surface area, which resulted in high catalytic activity/catalyst volume.

## Graphical abstract

Metallic iron particles loaded with aluminum hydride species and K^+^ species (AlH‐K+/Fe) in an appropriate manner, which is the inverse structure of supported metal catalysts adopted for many catalysts, increases the highly active sites for ammonia synthesis, which boosts the ammonia synthesis activity per catalyst surface area. This results in high ammonia synthesis activity per catalyst volume.

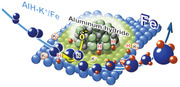

## 1 Introduction

Ammonia production by the Haber–Bosch (HB) process has increased the human population and supported modern civilization for over 100 years.^[^ ^1^ ^]^ However, more efficient ammonia production is now a significant concern for society, which has led to the search for other promising approaches. For example, the efficiency of ammonia synthesis from N_2_ and H_2_O by electrocatalysis has increased rapidly.^[^ ^2^, ^3^, ^4^ ^]^ With such approaches, the HB process remains attractive for the mass production of ammonia at present. A significant increase in the efficiency of the HB process established over many years has been a major challenge and requires heterogeneous catalysts that achieve high ammonia productivity under milder reaction conditions. In the HB process, where ammonia is produced by a catalyst‐filled reactor with a limited volume, the ammonia productivity in the reactor depends on the ammonia synthesis rate, not only per catalyst weight, but also per catalyst volume. For this reason, an increase in the ammonia synthesis rate/catalyst volume at low pressure and temperature would decrease energy consumption and significantly advance the HB process toward a sustainable future. The ammonia synthesis rate/catalyst weight, surface area and volume (rNH_3_w, rNH_3_s, and rNH_3_v) for the industrial promoted‐iron catalyst (Promoted‐Fe) composed of Fe, K (K_2_O), Al (Al oxides) and Ca (CaO) and those of new highly active catalysts based on Ru, Co, and Ni (300 °C, 0.9‐1.0 MPa) are summarized in Table S1 (Supporting Information).^[^ ^5^, ^6^, ^7^, ^8^, ^9^ ^]^ Promoted‐Fe was designed over 100 years ago by Mittasch and coworkers, and has remained at the forefront of mass ammonia production since the HB process was established.^[^ ^10^ ^]^ Table S1 (Supporting Information) indicates that some new transition metal (TM) catalysts have higher rNH_3_w than the iron catalyst. However, they could not cross the barrier of rNH_3_v for the classical Promoted‐Fe TM catalyst. This may be attributed to the structure of these new TM catalysts, i.e., supported metal structures. Supported metal catalysts with large specific surface areas tend to exhibit high rNH_3_w and have low density, so that a high rNH_3_w does not always result in a high rNH_3_v, although a high rNH_3_s can result in an increase in rNH_3_v.

As a first step to the development of desirable catalysts to achieve higher ammonia productivity with smaller energy consumption, we have adopted a catalyst design composed of metallic iron particles loaded with an appropriate promoter, which is the inverse structure of a supported metal catalyst. In many supported metal catalysts for ammonia synthesis, the highly active sites are expected to be limited to around the perimeter of the interface between the TM particles and the support material. In the inverse catalyst design, highly active sites can spread concentrically on the TM surface from the center of a deposited promoter. It has not been verified which structure is more effective in increasing rNH_3_s. An electron‐donating promoter on the catalyst structure is also a determinant factor for the ammonia synthesis activity. A N_2_ molecule is adsorbed on the TM surface by the donation of electrons from its bonding orbitals and acceptance of the electrons by its antibonding π^*^ orbitals (back‐donation). The back‐donation is enhanced by electron donors, which results in efficient N_2_ cleavage followed by ammonia formation. Potassium oxides such as K_2_O are considered to act as electron donors in Promoted‐Fe.^[^ ^11^, ^12^ ^]^ It was reported that alkaline earth metal hydrides and electrides in addition to Cs, Ba, and rare earth metal oxides are effective electron donors for Ru and Co.^[^ ^13^, ^14^, ^15^, ^16^, ^17^, ^18^, ^19^ ^]^ We recently reported that BaH_2_‐BaO mixture‐deposited iron nanoparticles on an electron‐donating CaH_2_ support (BaH_2_‐BaO/Fe/CaH_2_) act as an effective catalyst for low‐temperature ammonia synthesis, which suggests that iron combined with electron‐donating hydrides is a promising structure for catalytic ammonia synthesis.^[^ ^6^ ^]^ On the other hand, this supported iron catalyst was also inferior to Promoted‐Fe in terms of rNH_3_v as with other supported metal catalysts (Table S1, Supporting Information), and we have not achieved successful synthesis using only iron particles loaded with such hydride species, although it may have a high rNH3v.

## 2 Results and Discussion

### 2.1 Iron Particles Loaded with Al Species

**Figure 1:**
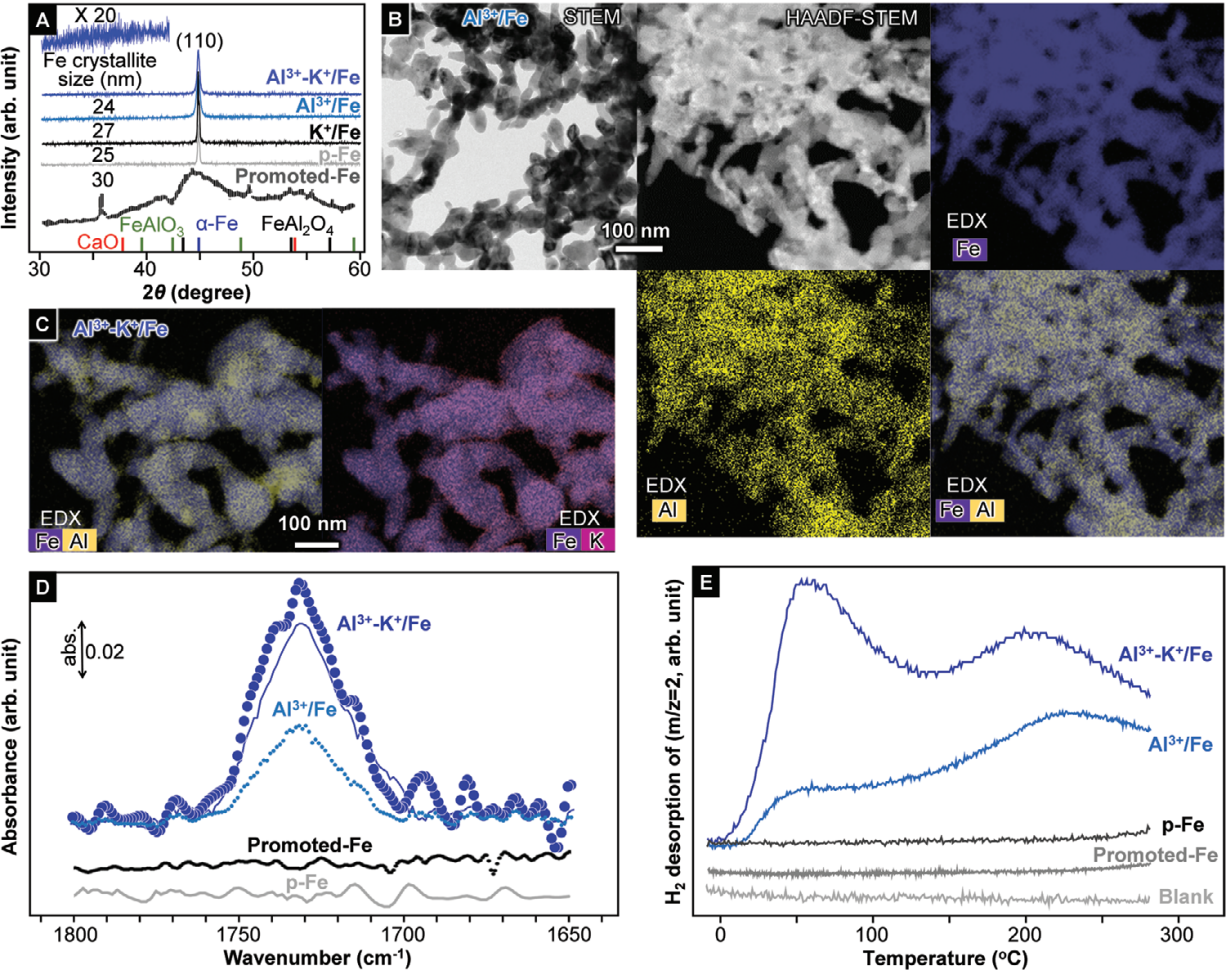
A) XRD patterns for the prepared iron‐based materials (Al^3+^‐K^+^/Fe (atomic ratio of Al/K/Fe = 2/2/96), Al^3+^/Fe (Al/Fe = 2/98), K^+^/Fe (K/Fe = 2/98), p‐Fe (Fe = 100)). Al^3+^‐K^+^/Fe, Al^3+^/Fe, and K^+^/Fe exhibited the highest catalytic activities for ammonia synthesis at respective atomic ratios of Al/K/Fe = 2/2/96, Al/Fe = 2/98, and K/Fe = 2/98. Further addition of Al^3+^ and/or K^+^ significantly decreased the catalytic performance. B) STEM, HAADF‐STEM, and EDX images of Al^3+^/Fe (Al/Fe = 2/98). C) EDX images for Al^3+^‐K^+^/Fe (Al/K/Fe = 2/2/96). D) FT‐IR spectra for Al^3+^/Fe, Al^3+^‐K^+^/Fe, P‐Fe, and Promoted‐Fe after ammonia synthesis. The samples were pressed into a disk in an Ar‐filled glove box after the ammonia synthesis reaction reached steady state. The disk in a sealed transmission FT‐IR cell was analyzed using a spectrometer in a flow of H_2_ (60 mL min^−1^) at 25 °C (solid blue (Al^3+^‐K^+^/Fe), dotted blue (Al^3+^/Fe), solid grey (p‐Fe), solid black (Promoted‐Fe) line spectra). The blue circle line spectrum was obtained by FT‐IR measurement of the α‐Fe_2_O_3_ precursor (for Al^3+^‐K^+^/Fe) reduced by H_2_ in the FT‐IR cell. The α‐Fe_2_O_3_ precursor and SiO_2_ powders were pressed into a double‐layered self‐supported disk (α‐Fe_2_O_3_/SiO_2_). The disk was placed in a cell that was connected to a closed gas‐circulation system equipped with a mass‐flow system and was then heated at 400 °C in a flow (60 mL min^−1^) of H_2_ under atmospheric pressure. After 24 h, the disk was cooled down to 25 °C and FT‐IR measurements were performed. E) H_2_‐TPD profiles for Al^3+^/Fe, Al^3+^‐K^+^/Fe, p‐Fe, and Promoted‐Fe after ammonia synthesis reached steady state (atmospheric pressure, 400 °C, 24 h).

Metallic iron particles containing groups 1, 2, and 13 cations were examined in this study. The evaporation of aqueous Fe(NO_3_)_3_ solution with dissolved additive cations, followed by calcination at 400 °C under atmospheric conditions formed α‐Fe_2_O_3_ with additive cations. Metallic iron particles were prepared by reduction of the resultant ferric oxide precursors in an ammonia synthesis reactor under a flow of H_2_ or under ammonia synthesis conditions (H_2_‐N_2_ flow) at 400 °C. Although many iron‐based materials that contain combinations of Al^3+^ and group 1 cation in addition to one type of cation from groups 1, 2, and 13 were prepared, only iron particles with Al^3+^ and both Al^3+^ and K^+^ (Al^3+^/Fe and Al^3+^‐K^+^/Fe) had higher catalytic performance for ammonia synthesis than Promoted‐Fe (see below). Al^3+^/Fe and Al^3+^‐K^+^/Fe exhibited the highest catalytic activities for ammonia synthesis at atomic ratios of Al/Fe = 2/98 and Al/K/Fe = 2/2/96. Further addition of Al^3+^ and/or K^+^ significantly decreased the catalytic performance. Powder X‐ray diffraction (XRD), X‐ray photoelectron spectroscopy (XPS), Fourier transform infrared (FT‐IR) spectroscopy, and temperature‐programmed desorption (TPD) measurements of these iron samples after H_2_ reduction or ammonia synthesis were performed using an Ar‐filled glove box to prevent exposure to the atmosphere. XRD profiles (**Figure** **1**A) for the resultant iron samples without any additives (p‐Fe) and with Al^3+^ (2.0 at%) and/or K^+^ (2.0 at%) (Al^3+^/Fe, K^+^/Fe, and Al^3+^‐K^+^/Fe) showed only a sharp diffraction peak due to α‐Fe(110). The Fe crystallite sizes shown in Figure 1 were estimated using the Scherrer equation with the α‐Fe (110) diffraction peak. XRD measurements revealed that all iron samples prepared in this study were very similar to the above four samples and were essentially distinct from H_2_‐reduced industrial iron catalysts such as those composed of metallic iron, FeAl_2_O_4_, FeAlO_3_, and CaO, as shown in Figure 1A.^[^ ^20^ ^]^ There was no significant difference in the microstructure observed using scanning transmission electron microscopy (STEM) among the prepared iron samples composed of large iron particles (> 30–50 nm) (Figure 1B). High‐angle annular dark field (HAADF)‐STEM and energy dispersive X‐ray spectroscopy (EDX) images for Al^3+^/Fe and Al^3+^‐K^+^/Fe (Figures 1B,C) showed large aggregated iron particles in which Al and K species were dispersed. XPS (Figure S1, Supporting Information) measurements indicated that the surfaces of Al^3+^/Fe and Al^3+^‐K^+^/Fe were composed of Al^3+^ and Fe species (atomic ratio of Al/Fe = 40/60) and Al^3+^, K^+^, and Fe species (Al/K/Fe = 35/22/43), and a considerable part of the Al^3+^ and K^+^ additives was segregated on the iron surfaces. The main component of surface Fe species on both samples was Fe^0^ (Fe^0^/Fe^2+^/Fe^3+^ = 58/28/13 (Al^3+^/Fe) and Fe^0^/Fe^2+^/Fe^3+^ = 58/30/12 (Al^3+^‐K^+^/Fe)). A notable feature of Figure S1 (Supporting Information) is that the Al 2p peaks for Al^3+^/Fe and Al^3+^‐K^+^/Fe were evident ≈74 eV. Taking into account the preparation of the iron samples and the ammonia synthesis conditions, metallic Al (Al^0^; 72.5–72.8 eV),^[^ ^21^, ^22^ ^]^ AlN (from 74.7 eV),^[^ ^23^ ^]^ and Al_2_O_3_ (75.4–75.8 eV)^[^ ^24^, ^25^ ^]^ are considered to be formed on the iron surfaces. However, the Al 2p peaks for Al^3+^/Fe and Al^3+^‐K^+^/Fe appeared apart from the binding energies of these Al species. The Al 2p peak of γ‐Al_2_O_3_ measured by the XPS used in this study is also shown in Figure S1 (Supporting Information) and is located at a somewhat larger binding energy than those of Al^3+^/Fe and Al^3+^‐K^+^/Fe, which suggests that the Al species on the iron material cannot be simply explained by only aluminum oxide species. The Al 2p peak of AlH_3_ was very recently reported to appear at 74.2 eV;^[^ ^26^ ^]^ aluminum hydride species may be considered as one of the candidates for Al^3+^ species on the iron materials.

FT‐IR spectroscopy was employed to clarify the Al species on the samples (Figure 1D). A vibrational band appeared at 1730 cm^−1^ in the spectra for Al^3+^/Fe (dotted blue) and Al^3+^‐K^+^/Fe (solid blue) after ammonia synthesis and in the spectrum for Al^3+^‐K^+^/Fe (blue circle line) obtained by H_2_ reduction in an FT‐IR cell. This band was not observed in the spectra for p‐Fe and Promoted‐Fe after the ammonia synthesis reaction. An FT‐IR spectrum with a wide wavenumber range for Al^3+^‐K^+^/Fe after reaction is shown in Figure S2 (Supporting Information), which shows a band at ≈900 cm^−1^ in addition to a band at 1730 cm^−1^. No bands due to COO^−^ and CO_3_^2−^ (1400 cm^−1^) were observed at 1400–1450 cm^−1^ in the FT‐IR spectrum. Molecular AlH_3_ was reported to have a νAl‐H band at 1710 cm^−1^ and two δAl‐H bands at 800 and 900 cm^−1^.^[^ ^27^ ^]^ In the case of solid AlH_3_ ((AlH_3_)_n_), two broadbands were observed at 1500–1900 cm^−1^ (νAl‐H) and 500–1000 cm^−1^ (δAl‐H).^[^ ^28^ ^]^ An FT‐IR spectrum for KAlH_4_ shows several broadbands (1750–1785 cm^−1^ due to νAl‐H, and 820 and 900 cm^−1^ due to two δAl‐H modes).^[^ ^29^ ^]^ From these FT‐IR results, including Figure 1D and S2 (Supporting Information), one possibility for the Al species formed on Al^3+^/Fe and Al^3+^‐K^+^/Fe is aluminum compounds with Al─H bonds such as aluminum hydrides. There seems to be no significant difference in νAl‐H and δAl‐H between AlH_3_ and AlH_4_^−^; therefore, we have yet to clarify the details of the aluminum hydride species on the iron surfaces. A further increase in the Al content in AlH/Fe and AlH‐K^+^/Fe significantly decreased the catalytic performance as mentioned above. The XRD profiles, and FT‐IR and Al 2p XPS spectra for AlH‐K^+^/Fe samples of Al/K/Fe = 2/2/96 and 20/2/78 are shown in Figure S3 (Supporting Information). The latter, which had a larger amount of Al than the former, showed lower catalytic activity than the former, similar to the ammonia synthesis rate for Promoted‐Fe (see below). Figure S3 (Supporting Information) shows that AlN is formed on the latter without the species with the vibrational mode ≈1700 cm^−1^. The Al 2p peak for AlH‐K^+^/Fe (Al/K/Fe = 20/2/78) was located at 74.7 eV, which does not contradict that of AlN (74.7 eV) and is larger than that (≈74 eV) of AlH‐K^+^/Fe (Al/K/Fe = 2/2/96). This indicates that the surface species with the vibrational mode ≈1700 cm^−1^ in FT‐IR are closely related to those with the Al 2p peak around 74 eV on AlH‐K^+^/Fe (Al/K/Fe = 2/2/96).

H_2_‐TPD (Figure 1E) measurements for the samples used to synthesize ammonia for over 40 h in a flow of H_2_ and N_2_ (400 °C, 0.9 MPa) indicated that H_2_ is desorbed from Al^3+^/Fe and Al^3+^‐K^+^/Fe over a wide temperature range from room temperature. Hydrogen adatoms on a metallic iron surface desorb as H_2_, even below room‐temperature;^[^ ^30^ ^]^ no H_2_ desorption peak was observed on Promoted‐Fe and p‐Fe under the experimental conditions. Under the assumption that Al^3+^‐K^+^/Fe is composed of uniform spherical particles with a known diameter and H is bonded only to surface Al, the atomic ratio of H to Al on the sample surface (H:Al) was estimated to be 2.8:1.0 from H_2_‐TPD measurements (Discussion S1, Supporting Information). A considerable amount of surface Al atoms was therefore estimated to form AlH_3_ if the surface Al species consisted of only AlH_3_. These results are consistent with the formation of the aluminum hydride species on Al^3+^/Fe and Al^3+^‐K^+^/Fe, so that Al^3+^/Fe and Al^3+^‐K^+^/Fe were denoted as AlH (aluminum hydride species)/Fe and AlH‐K^+^/Fe, respectively. On the other hand, the iron samples prepared without Al^3+^ neither had the νAl‐H band in the FT‐IR spectrum nor large H_2_ desorption peaks, as also observed for p‐Fe. From these results, the AlH/Fe and AlH‐K^+^/Fe prepared in this study are expected to consist of Al species deposited on large metallic iron particles that themselves consist of 20–30 nm α‐Fe aggregates. Al^3+^ species with Al─H bonds on AlH/Fe and AlH‐K^+^/Fe account for 40% and 35% of the surface atoms, respectively. In the case of AlH‐K^+^/Fe, ≈20% of the surface atoms were K^+^ species.

### 2.2 Catalytic Performance of Iron Particles Loaded with Aluminum Hydride Species

**Figure 2:**
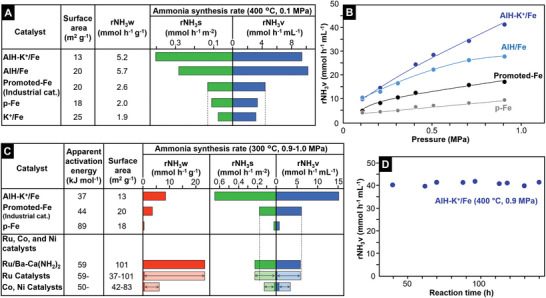
A) Ammonia synthesis rate for each iron‐based catalyst (0.1 MPa, 400 °C). B) Correlation of ammonia synthesis rate with pressure for each iron‐based catalyst (400 °C). C) Ammonia synthesis activities of various catalysts at 300 °C (0.9–1.0 MPa, WHSV: 36 000 mL g^−1^ h^−1^). Details of these catalysts, including numerical data, are given in Table S1(Supporting Information). D) Time course of the ammonia synthesis rate over AlH‐K^+^/Fe at 300 °C (0.9 MPa).

**Figure** **2**A shows rNH_3_w, rNH_3_s, and rNH_3_v for the tested iron samples (400 °C, 0.10 MPa). The catalytic activities of all the prepared iron samples, except AlH/Fe and AlH‐K^+^/Fe, were lower than that of Promoted‐Fe, as shown in the results for p‐Fe and K^+^/Fe; group 1 and 2 cations such as K^+^ and Ba^2+^ could not act as effective promoters in this study, although it is well known that the addition of these cations to catalytic systems accelerates ammonia synthesis activity.^[^ ^5^, ^14^, ^17^, ^31^, ^32^, ^33^ ^]^ Alkaline metal oxides such as K_2_O and Cs_2_O obtained by the thermal decomposition of nitrates or carbonates over 600 °C were reported to enhance ammonia synthesis over Promoted‐Fe and supported iron catalysts, as electron donors.^[^ ^11^, ^12^, ^34^ ^]^ However, the formation of such oxide species is not expected in this study where the iron catalysts were prepared at 400 °C to prevent the sintering of the metallic iron particles. It was confirmed that a further increase in the precursor preparation temperature and/or the precursor reduction temperature beyond 500 °C decreased the ammonia synthesis rates for iron catalysts, even if electron‐donating alkaline metal oxides were formed on the iron catalysts. This is due to significant sintering that reduces the specific surface area below 3 m^−2^ g^−1^, which negates the increase in catalytic activity by electron donors on the iron catalysts without structural materials such as Al_2_O_3_ in Promoted‐Fe and support materials in supported iron catalysts that prevent sintering of the metallic iron particles; therefore, it is difficult for alkaline metal oxides to enhance the catalytic activity of the catalyst structure adopted in this study. In contrast, all the activity indices for AlH/Fe and AlH‐K^+^/Fe were more than double those for Promoted‐Fe; both catalysts could first synthesize ammonia at more than twice the rNH_3_v for the conventional industrial iron catalyst. Taking into account the low catalytic activity of K^+^/Fe, the high activities of AlH/Fe and AlH‐K^+^/Fe are considered to be due to the aluminum species rather than K^+^.

While AlH/Fe had similar activity to AlH‐K^+^/Fe at 0.10 MPa, the latter exceeded the former at 0.2 MPa, and the difference in activity between the two catalysts increased with the pressure (Figure 2B). To understand the difference in catalysis between AlH/Fe and AlH‐K^+^/Fe, the reaction orders in the rate equation for these catalysts were measured at 400 °C under 0.9 MPa (Discussion S2, Supporting Information). If we assume that the ammonia synthesis rate (rNH_3_w) is expressed by r = *k* (*P*N_2_)^α^(*P*H_2_)^β^(*P*NH_3_)^γ^ (*k*; rate constant), then a relative reaction order relationship between H_2_ and N_2_ can be expected. The H_2_ reaction orders (β) for AlH/Fe and AlH‐K^+^/Fe were estimated to be 3.4 and 7.6, respectively. There was no significant difference in the N_2_ reaction order (γ) between the catalysts (1.5–1.7), which indicates that the catalytic activity increment to H_2_ pressure increment in AlH/Fe is more than three orders of magnitude lower than that of AlH‐K^+^/Fe. This resembles so‐called hydrogen poisoning, where preferential dissociative H_2_ adsorption to dissociative N_2_ adsorption on TM surfaces prevents N_2_ cleavage that would lead to ammonia synthesis.^[^ ^6^, ^35^ ^]^ Iron has been considered as a TM that prevents hydrogen poisoning. However, iron is also susceptible to hydrogen poisoning as long as the dissociative adsorption of H_2_ is in equilibrium with H_2_ desorption over the iron surface.^[^ ^36^ ^]^ Ertl *et al.* reported that K^+^ on Fe(110) and (111) surfaces halved the sticking coefficient for dissociative H_2_ adsorption,^[^ ^37^ ^]^ which suggests that K^+^ lowers the negative effect for ammonia synthesis with an increase of the H_2_ pressure over AlH‐K^+^/Fe. The correlation of rNH_3_v with the pressure over AlH‐K^+^/Fe and Promoted‐Fe (400 °C) below 5 MPa is shown in Figure S4 (Supporting Information). AlH‐K^+^/Fe also exhibited higher catalytic performance than Promoted‐Fe at 5 MPa. Figure 2C compares AlH‐K^+^/Fe with other catalysts, including Ru, Co, and Ni‐deposited support materials that were recently reported as highly active catalysts, at 300 °C under 0.9–1.0 MPa.^[^ ^5^, ^7^, ^8^, ^9^ ^]^ A decrease in the synthesis temperature of ammonia produced through exothermic reaction increases the theoretical ammonia yield while decreasing the pressure, which is reconciled with an increase in the ammonia yield and a decrease in energy consumption for heating and pressurization.^[^ ^6^ ^]^ On the other hand, a low reaction temperature enhances the lack of ammonia synthesis due to hydrogen poisoning.^[^ ^6^ ^]^ The rNH_3_v for AlH‐K^+^/Fe reached ca. three times that for Promoted‐Fe, even though the catalyst had the smallest surface area. A decrease in reaction temperature further increased the difference in rNH_3_v and rNH_3_w. Figure 2C shows that rNH_3_v was not correlated with rNH_3_w but with rNH_3_s, and AlH‐K^+^/Fe had much higher rNH_3_s than the other catalysts. Arrhenius plots for promoted‐Fe and AlH‐K^+^/Fe are shown in Figure S5 (Supporting Information). There was no significant difference in slope due to the apparent activation energy, but there was a significant difference in the ln(ammonia synthesis rate)‐intercept derived from the pre‐exponential factor including the entropy term between the two Arrhenius plots. This can be interpreted as an increase in active sites available for the reaction on AlH‐K^+^/Fe, despite the small specific surface area. This enhances the rNH_3_s for AlH‐K^+^/Fe. C7 (Fe^0^ atoms with seven nearest neighbors) sites have been proposed as effective sites for ammonia synthesis on metallic iron surfaces.^[^ ^38^, ^39^ ^]^ Such sites themselves cannot exhibit high catalytic performance without electron‐donating promoters,^[^ ^40^, ^41^ ^]^ as shown in the catalytic activity of metallic iron particles without any additives (p‐Fe), because they do not have strong electron‐donating capability. Strong electron donation from promoters to the iron surfaces therefore increases the highly active sites with strong electron‐donating capability. p‐Fe prepared in a similar manner to AlH‐K^+^/Fe was similar to AlH‐K^+^/Fe in terms of particle and crystallite size, but with more Fe^0^ atoms than the latter (Figure S1, Supporting Information). This implies that p‐Fe has a larger amount of Fe^0^ atoms to synthesize ammonia than AlH‐K^+^/Fe. Nevertheless, p‐Fe has lower catalytic performance than AlH‐K^+^/Fe due to the lack of a promoter to enhance the electron‐donating capability of the sites. Taking into account these results, it is expected that iron combined with aluminum hydride species increases the active sites with strong electron‐donating capability on the catalyst structure, even with a limited surface area. It has not been reported that aluminum hydride species act as strong electron donors for TM. The Arrhenius plots also indicate that there is no difference in the apparent activation energy of the active sites between Promoted‐Fe and AlH‐K^+^/Fe. We do not have a satisfactory explanation for this phenomenon. There may be a limit to the enhancement of the active sites by electron donors on iron, which has a low electron affinity (see below).

**Figure 3:**
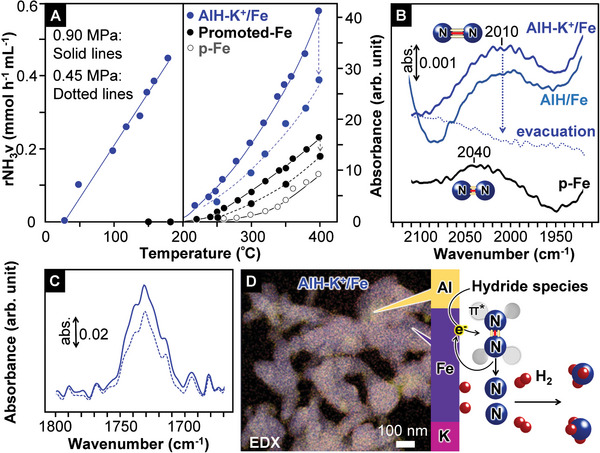
A) Correlation of ammonia synthesis rate with temperature for iron‐based catalysts. After no increase or decrease in activity was observed for over 20 h (400 °C, 0.9 MPa), the catalyst was cooled down to below 20 °C in a flow of N_2_ (60 mL min^−1^) and held in this flow for 5 h. After no ammonia formation was confirmed, the catalyst was heated at specific temperatures in a flow of N_2_–H_2_. B) FT‐IR spectra for ^14^N_2_‐adsorbed p‐Fe, AlH/Fe, and AlH‐K^+^/Fe. Each α‐Fe_2_O_3_ precursor double‐layered self‐supported disk (α‐Fe_2_O_3_/SiO_2_) in the FT‐IR cell was heated for 24 h at 400 °C in a flow (60 mL min^−1^) of N_2_–H_2_ (N_2_:H_2_ = 1:3) under atmospheric pressure, which resulted in the formation of p‐Fe, AlH/Fe, and AlH‐K^+^/Fe, followed by cooling to 25 °C. After the FT‐IR cell was evacuated under vacuum (< 1.3 Pa), N_2_ (53 kPa) was introduced into the cell at 25 °C. The broadband observed at 2010 cm^−1^ for AlH‐K^+^/Fe in the presence of N_2_ disappeared by vacuum evacuation of the FT‐IR cell. C) FT‐IR spectra for AlH‐K^+^/Fe at 25 °C (solid line) and 300 °C (dashed line) after 10 h in a flow of N_2_ and H_2_ under 0.1 MPa (ammonia synthesis conditions). There was no significant difference in the spectra among the disks after the reaction for 1, 10, and 20 h. D) EDX composite image for AlH‐K^+^/Fe and schematic of the reaction mechanism for ammonia synthesis over AlH/Fe and AlH‐K^+^/Fe.

AlH‐K^+^/Fe produced ammonia without any decrease in activity, even after reaction for over 100 h (Figure 2D). The XPS spectrum and XRD profile for AlH‐K^+^/Fe remained unchanged after the reaction (Figure S6, Supporting Information), which clearly indicates that AlH‐K^+^/Fe acts as a stable catalyst for ammonia synthesis. The small apparent activation energy (E_a_ = 37 kJ mol^−1^) and the high rNH_3_v of AlH‐K^+^/Fe in Figure 2C imply that AlH‐K^+^/Fe can act as an effective catalyst, even at lower temperatures. Figure 3A shows that ammonia synthesis over AlH‐K^+^/Fe began at 50 °C. This was also confirmed by direct mass spectrometry (Figure S7, Supporting Information), and there was no difference in the ammonia synthesis rate obtained by direct mass spectrometry and ion chromatography which is typically used for analysis.^[^ ^42^ ^]^ All catalysts, except for the Ru nanoparticle‐deposited CaFH solid solution, did not work for the reaction at such a low temperature.^[^ ^42^ ^]^ In addition, the iron catalyst was superior to Promoted‐Fe in terms of rNH_3_v up to 400 °C. The ammonia synthesis rate of Promoted‐Fe at 400 °C under 0.9 MPa was similar to that of AlH‐K^+^/Fe at 310 °C under 0.9 MPa and was also achieved by AlH‐K^+^/Fe at 345 °C under 0.45 MPa. The iron catalyst thus exhibits higher ammonia productivity, even at lower temperatures and pressure than the industrial iron catalyst (**Figure** **3**A).

### 2.3 Ammonia Formation Over Iron Particles Loaded with Aluminum Hydride Species

The high ammonia productivity of the iron catalyst at low temperatures can be attributed to many active sites with a small E_a_ (37 kJ mol^−1^). The E_a_ of p‐Fe without any electron donors exceeded 80 kJ mol^−1^ (Figure 2C). The cleavage of N_2_ molecules is considered to be the rate‐determining step over many ammonia synthesis catalysts; therefore, the enhancement of electron donation into the anti‐bonding π^*^ orbitals of adsorbed N_2_ via TMs facilitates N_2_ cleavage. The small E_a_ of AlH‐K^+^/Fe is therefore indicative of the strong electron‐donating capability of the iron surface with the aluminum hydride species, which has not been considered to date. The iron surfaces were examined using FT‐IR spectroscopy with N_2_ as a probe molecule (Figure 3B). ^14^N_2_‐adsorbed p‐Fe showed a broadband due to N≡N stretching (νN_2_) at 2040 cm^−1^, which was consistent with the high‐resolution electron energy loss spectroscopy (HREELS) results for an N_2_‐adsorbed Fe(110) single‐crystal surface, although the νN_2_ band for N_2_ adsorbed on a metallic iron surface has not yet been observed using infrared spectroscopy.^[^ ^43^ ^]^ In the case of AlH/Fe and AlH‐K^+^/Fe, a broadband assignable to νN_2_ in the FT‐IR spectra of the ^15^N_2_‐adsorbed iron catalysts (Figure S8, Supporting Information) appeared ≈2010 cm^−1^. The red‐shift indicates that N_2_ on AlH/Fe and AlH‐K^+^/Fe accept stronger electron donation and are more dissociative than N_2_ on p‐Fe. Iron has a considerably lower electron affinity (15 kJ mol^−1^) than other TMs (64‐112 kJ mol^−1^) employed for ammonia synthesis. Therefore, promoters with large negative charges would be required to enhance the electron‐donating capability on iron. One possible explanation for this is the aluminum hydride species. Density functional theory (DFT) calculations for an aluminum hydride cluster ((AlH_3_)_28_) revealed that the partial removal of H atoms from an (AlH_3_)_28_ cluster, which originally has negative charges, further increases the negative charges until the amount of H^–^ ions is halved.^[^ ^44^ ^]^ The partial removal of H atoms from metal hydrides, such as CaH_2_, BaH_2_, and CaFH, combined with TMs has also been reported to result in strong electron donation to the TMs, which significantly enhances the ammonia synthesis activity.^[^ ^6^, ^13^, ^14^, ^42^ ^]^ To estimate the concentration of H^–^ in the Al species on iron during the reaction, FT‐IR spectra for AlH‐K^+^/Fe were measured at 25 and 300 °C in a flow of H_2_ and N_2_ under atmospheric pressure (Figure 3C). Ammonia formation was not observed over the catalyst at 25 °C under these conditions. The intensity of the νAl‐H band at 1730 cm^−1^ under ammonia synthesis conditions (300 °C) was somewhat smaller (≈75%) than that at 25 °C; a major part of H^–^ thus remains in the aluminum species on the iron surface. The DFT results indicate that such aluminum hydride species with a certain degree of H^−^ defects would have high electron‐donating capability. Heating aluminum hydride generally removes all H^−^ as H_2_, which results in the formation of metallic aluminum without strong electron‐donating power. H^–^ in aluminum species that possess a considerable amount of H^−^ under the reaction conditions would be in equilibrium with H_2_ in the gas phase though the metallic iron surface.

As a summary of these results, the mechanism postulated for ammonia synthesis over the iron catalyst in this study is shown schematically in Figure 3D. H_2_‐reduction of α‐Fe_2_O_3_ precursors that contain Al^3+^ and both Al^3+^ and K^+^ in an ammonia synthesis reactor readily form metallic iron particles (> 30–50 nm). Aluminum hydride species and both aluminum hydride and K^+^ species are segregated on the AlH/Fe and AlH‐K^+^/Fe surfaces. From the H_2_‐TPD profiles (Figure 1E) and FT‐IR spectra (Figure 1D) measured for AlH/Fe and AlH‐K^+^/Fe, partial removal of H atoms from the aluminum hydride species is considered to enable strong electron donation to the N_2_ molecules adsorbed on the iron surface, which facilitates N_2_ cleavage and ammonia synthesis (Figure 3D). Therefore, while AlH/Fe and AlH‐K^+^/Fe have higher catalytic activities than Promoted‐Fe (Figure 2A), AlH/Fe is inferior to AlH‐K^+^/Fe in terms of the ammonia synthesis rate with an increase in pressure because the activity increment per H_2_ pressure increment for AlH/Fe is considerably lower than that for AlH‐K^+^/Fe. One possibility is that H_2_ adsorption prevents dissociative N_2_ adsorption on AlH/Fe due to hydrogen‐poisoning. On the other hand, ammonia synthesis over AlH‐K^+^/Fe is less affected by an increase in H_2_ pressure than that over AlH/Fe, which suggests that the addition of K^+^ species on the catalyst surface lowers the negative effect for ammonia synthesis by H_2_ pressurization; K^+^ may prevent excess dissociative H_2_ adsorption. AlH‐K^+^/Fe can synthesize ammonia even at 50 °C and exhibits high catalytic performance in the low‐temperature range because the H_2_‐TPD profile and large positive H_2_ reaction order for AlH‐K^+^/Fe indicate that the aluminum hydride species has strong electron‐donating capability above room‐temperature and AlH‐K^+^/Fe is less affected by the prevention of ammonia synthesis with an increase in H adatoms as observed with hydrogen‐poisoning. Although metallic iron particles loaded with group 1 and 2 cations, which have the same catalyst structure as AlH/Fe and AlH‐K^+^/Fe, have a larger amount of surface Fe^0^ atoms effective for ammonia synthesis than AlH/Fe and AlH‐K^+^/Fe (Figure S1, Supporting Information), and thus lower catalytic performance than Promoted‐Fe because these cations do not act as strong electron donors on the catalyst structure.

## 3 Conclusion

Metallic iron particles loaded with appropriate promoters such as Al^3+^ and K^+^ species, which is the inverse structure of supported metal catalysts adopted for many catalysts, increase the highly active sites for ammonia synthesis, which boosts the ammonia synthesis rate per catalyst surface area (rNH_3_s). This results in a high ammonia synthesis rate per catalyst volume. Strong electron donation to N_2_ through the metallic iron surface in the iron catalyst facilitates the cleavage of N_2_ molecules, which is the rate‐determining step over many ammonia synthesis catalysts. The partial removal of H atoms from Al species with Al^3+^‐H^−^ bonds possibly imparts strong electron‐donating capability to the iron surface, which has a low electron affinity. The catalyst design adopted in this study may also be advantageous to increase rNH_3_v, due to the high density and rNH_3_s. The performance of the proposed iron catalyst obtained from ubiquitous, abundant, and inexpensive iron without special atmosphere or equipment demonstrates that iron, which was first found for the Haber‐Bosch process by Mittasch and coworkers over 100 years ago, is still an attractive material for ammonia synthesis.

## 4 Experimental Section

### Catalyst Preparation and Catalytic Activity Evaluation

As the first step to prepare metallic iron particle catalysts with additives, group 1, 2, and 13 nitrates, 7.5 g of Fe(NO_3_)_3_·9H_2_O (Kanto Chemical) and 2.1 g of trifluoroacetic acid were dissolved in 30 mL of distilled water, which was then removed from the solution by rotary evaporation at 70 °C. In the preparation of metallic iron particles without any additives (p‐Fe), water was removed from an aqueous solution obtained in the same way by dissolving 7.5 g of Fe(NO_3_)_3_·9H_2_O and 2.1 g of trifluoroacetic acid in 30 mL of distilled water. The addition of trifluoroacetic acid ensured the reproducible preparation of the highly active iron catalysts. The remaining solid was heated at 450 °C for 10 h in the air to yield red α‐Fe_2_O_3_ powder with or without groups 1, 2, and 13 cations. The iron powder catalyst was obtained by heating α‐Fe_2_O_3_ precursor powder in a stainless steel fixed‐bed reactor^[^ ^6^ ^]^ (Fe_2_O_3_ precursor; ≈0.14 g (iron catalyst; ≈0.1 g)) at 400 °C in a flow of N_2_–H_2_ (N_2_ and H_2_ >99.9999%, N_2_/H_2_ = 1:3, 60 mL min^−1^, weight hourly space velocity (WHSV): 36000 mL g cat^−1^ h^−1^) under atmospheric pressure. After ammonia synthesis reached the steady state (within 24 h), the iron powder catalyst in the reactor was examined under various ammonia synthesis conditions. In the case of preparation of the iron pellet catalyst, the Fe_2_O_3_ precursor powder was heated at 400 °C in a flow of H_2_ in a tubular electric furnace (H_2_ >99.9999%, 60 mL min^−1^). After several hours, the resultant metallic iron powder was cooled down to room‐temperature and was then passivated by exposure to trace air. The passivated metallic iron powder was pressed (300 MPa) into pellets (1.6 mm diameter, 1.0–2.0 mm thick) under atmospheric conditions. These pellets were heated in another stainless steel fixed‐bed reactor (catalyst ≥0.5 g) under a flow of N_2_–H_2_ in a similar manner. It took ≈24 h for ammonia synthesis to reach a steady state. There was no significant difference in the apparent density among the prepared pellets. To evaluate the catalytic performance of the current industrial iron catalyst (Promoted‐Fe) for ammonia production, pellets of an unreduced industrial iron catalyst, which was similar to a magnetite‐based precursor containing K, Al, and Ca oxides used in a previous report,^[^ ^20^ ^]^ or the powder obtained by grinding the pellets, were pretreated according to an industrial activation procedure. Unreduced Promoted‐Fe in the stainless steel fixed‐bed reactors was heated at 200 °C (heating rate 5 °C min^−1^) for 5h under H_2_ flow. The reactor temperature was raised to 400 °C at a rate of 1.7 °C h^−1^ under H_2_ flow and was held at this temperature in a flow of N_2_–H_2_ (N_2_/H_2_ = 1:3, 60 mL min^−1^) under atmospheric pressure. After it was confirmed that the ammonia synthesis rate in a steady state was within ± 5% of a specified value shown in the procedure, the pellets and powder were examined under various ammonia synthesis conditions. Ru‐, Co‐, and Ni‐containing catalysts were prepared according to previously reported methods.^[^ ^5^, ^7^, ^8^ ^]^ The specific surface areas of the prepared catalysts were consistent with those of the previous reports.

The catalysts in the reactors were evaluated at 20–400 °C under a flow of N_2_–H_2_ (N_2_ and H_2_, >99.9999%, N_2_:H_2_ = 1:3, 60 mL min^−1^, WHSV: 36000 mL g cat^−1^ h^−1^) at 0.1–0.9 MPa. The ammonia produced was trapped in 5 mm H_2_SO_4_ aqueous solution and the amount of NH_4_^+^ generated in the solution was estimated using an ion chromatograph (LC‐2000 plus, Jasco) equipped with a thermal conductivity detector. The rate of ammonia formation was repeatedly measured more than 3 times after the ammonia formation rate remained constant for over 1 h. It was verified that the measured rate of ammonia formation had an error of less than 5%. The ammonia synthesis rate was also measured by direct mass spectrometry (M‐101QA‐TDM, Canon Anelva, Japan). There was no difference in the ammonia synthesis rate measured by direct mass spectrometry or ion chromatography. The rNH_3_v, ammonia synthesis rate/catalyst volume, of each catalyst, was estimated as described in Table S1 (Supporting Information).

### Characterization

XRD patterns were obtained using Cu Kα radiation (Miniflex600C, Rigaku). The catalysts after ammonia synthesis reached the steady state (atmospheric pressure, 400 °C) were transferred from the reactor to a sealed XRD sample holder in an Ar‐filled glove box and XRD measurements were then performed. Nitrogen adsorption–desorption isotherms were measured at −196 °C with a surface‐area analyzer (BELSORP‐mini ΙΙ, MicrotracBEL) to estimate the Brunauer–Emmett–Teller (BET) surface areas. The morphologies of the samples were observed using STEM, HAADF‐STEM, and EDX (JEM‐ARM 200F, Jeol). H_2_‐TPD profiles were measured by heating (1 °C min^−1^) a sample (≈100 mg) in a flow of Ar (30 mL min^−1^), and the concentration of H_2_ was monitored with a quadrupole mass spectrometer (BELMass, MicrotracBEL, Japan). Transmission FT‐IR spectra were measured by two methods using a spectrometer (FT/IR‐6100, Jasco) equipped with a mercury–cadmium–tellurium detector at a resolution of 4 cm^−1^. The catalyst powders in the reactors after ammonia synthesis reached the steady state (atmospheric pressure, 400 °C) were pressed into self‐supported disks in an Ar‐filled glove box without exposure to air. A disk was placed in a sealed quartz cell equipped with NaCl windows in the Ar‐filled glove box, and the cell connected to a closed gas‐circulation system was placed in the spectrometer. FT‐IR spectra for the iron catalysts obtained by the reduction of an α‐Fe_2_O_3_ precursor in an FT‐IR quartz cell were also measured in this study. The α‐Fe_2_O_3_ precursor and SiO_2_ powders (Q‐10, Fuji Silysia Chemical Ltd.) were pressed into a double‐layered self‐supported disk (α‐Fe_2_O_3_/SiO_2_) under atmospheric conditions. The disk was placed in a cell that was connected to a closed gas‐circulation system equipped with a mass‐flow system, and was heated at 400 °C in a flow (60 mL min^−1^) of H_2_ under atmospheric pressure. This formed metallic iron particles on the SiO_2_ support disk. After 24 h, FT‐IR spectra of the metallic iron particles were measured under various conditions (^14^N_2_, ^15^N_2_, and H_2_ under vacuum evacuation or at 0.1 MPa). XPS (ESCA‐3200, Shimadzu, Mg K*α*, 8 kV, 30 mA) measurements were performed in conjunction with an Ar‐filled glove box, where the samples were moved to the ultra‐high vacuum XPS apparatus through the Ar‐filled glove box without exposure to the ambient air. The binding energy was corrected with respect to the Au 4f_7/2_ peak of Au‐deposited samples.

## Conflict of Interest

The authors declare no conflict of interest.

## Supporting information



Supporting Information

## Data Availability

The data that support the findings of this study are available from the corresponding author upon reasonable request.
