# Supplementary material for: Ammonia Synthesis Over an Iron Catalyst with an Inverse Structure
Source: Adv Sci (Weinh). 2025 Jan 23;12(11):2410313. doi: 10.1002/advs.202410313 (PMC11923926; doi:10.1002/advs.202410313)
Supplement: Supplementary file 1 — Supporting Information [file ADVS-12-2410313-s001.docx]

Supplementary Information for

**Ammonia Synthesis over An Iron Catalyst with An Inverse Structure**

Masashi Hattori^1^, Kento Miyashita^1^, Yuki Nagasawa^1^,

Ryo Suzuki^2^ & Michikazu Hara^1⋆^

^1^Materials and Structures Laboratory, Institute of Integrated Research, Institute of Science Tokyo, 4259 Nagatsuta, Midori-ku, Yokohama 226–8503, Japan.

^2^ F.C.C. CO., LTD., 7000-36 Nakagawa, Hosoe-cho, Hamana-ku, Hamamatsu, Shizuoka, 431-1304, Japan

E-mail: [mhara@msl.titech.ac.jp](mailto:mhara@msl.titech.ac.jp)

Contents

1. Supplementary discussion

1. Estimation of the amount of aluminum hydride species on AlH-K^+^/Fe

2. Reaction orders for each iron-based catalyst

2. Supplementary data

Table 1-2, Figures 1-8

**1. Supplementary discussion**

**Discussion S1. Estimation of the amount of aluminum hydride species on AlH-K^+^/Fe**

Let us assume that AlH-K^+^/Fe is composed of uniform spherical particles (without inner pores) with a radius (*r*), then *r*, weight, surface area, and the number of surface Fe atoms of one catalyst particle are estimated to be 2.93×10^-8^ m, 8.29×10^-16^ g, 1.08×10^-14^ m^2^, and 1.86×10^5^, respectively, from the true density of Fe (7.87×10^6^ g m^-3^) and Fe atoms per bcc Fe(110) surface (1.72×10^19^ m^-2^). The estimated *r* is consistent with the EDX images for AlH-K^+^/Fe in Fig. 2C. The Al atoms account for ca. 40% of the particle surface atoms (XPS); therefore, the surface Al atoms are expected to be 7.44×10^4^ per AlH-K^+^/Fe particle. H_2_-TPD analysis to remove all H atoms in a sample by an increase in the temperature to above 800 °C revealed that 0.1 g of AlH-K^+^/Fe releases 2.1×10^-5^ mol of H_2_. As a result, one AlH-K^+^ particle contains 2.1×10^5^ H atoms which is ca. 3 times the surface Al atoms.

**Discussion S2. Reaction orders for each iron-based catalyst**

Let us assume that the ammonia synthesis rate (rNH_3_w) is expressed by the following equation, then the relative reaction order relationship between H_2_ and N_2_ can be estimated from α and β in the equation.

rNH_3_w = *k* (*P*N_2_)^α^ (*P*H_2_)^β^ (*P*NH_3_)^γ^

where *k* is the rate constant.

The reaction orders with respect to N_2_ and H_2_ were obtained at a constant flow rate (60 mL min^-1^) using Ar gas (purity > 99.99999%) as a diluent, and that for NH_3_ was determined with a 3H_2_+N_2_ mixture (0.9 MPa) by changing the synthesis gas flow rate.　Reaction orders over each iron catalyst are shown in Table S2.

**2. Supplementary data**

**Table S1.** Ammonia synthesis activities for various catalysts (300 °C, 0.9-1.0 MPa, WHSV: 36000 mL g cat^–1^ h^-1^)

| Catalyst | Surface area  (m^2^ g^-1^) | Catalytic activity  rNH_3_w^a^ 　 rNH_3_s^b^ 　 rNH_3_v (mmol h^-1^ g^-1^)(mmol h^-1^ m^-2^) (mmol h^-1^mL^-1^) | | | Reference |
| --- | --- | --- | --- | --- | --- |
| AlH-K^+^/Fe^c^ | 13 | 8.5 | 0.65 | 15.2 | This work |
| AlH/Fe^c^ | 20 | 2.5 | 0.13 | 4.5 | This work |
| p-Fe^c^ | 18 | 0.5 | 0.03 | 0.8 | This work |
| Promoted-Fe^c^ | 20 | 3.6 | 0.18 | 6.0 | This work |
| BaH_2_-BaO/Fe/CaH_2_^c^ | 18 | 3.7 | 0.21 | 3.6 | This work, 1 |
| Cs-Ru/MgO^d^ | 92 | 0.6 | 0.01 | 0.3 | 2 |
| Ru/Ba-Ca(NH_2_)_2_^d^ | 101 | 23.3 | 0.23 | 5.8 | 2 |
| Co/Ba-Ca(NH_2_)_2_^d^ | 83 | 6.2 | 0.07 | 1.6 | 2 |
| Co@BaO/MgO^d,e^ | 42 | 5.3 | 0.13 | 2.5 | 3 |
| Ni/LaN^d^ | 43 | 0.67 | 0.02 | 0.6 | 4 |
| Ni/CeN^d^ | 47 | 3.7 | 0.08 | 3.4 | 5 |

^a^ rNH_3_w was obtained for the powder catalyst.

^b^ rNH_3_s was calculated from rNH_3_w and the specific surface area.

^c^ rNH_3_v for each iron-based catalyst, including promoted-Fe, was obtained by ammonia synthesis over the catalyst pellets. The apparent catalyst pellet volume was estimated from the catalyst pellets after reaction.

^d^ rNH_3_v for Ru-, Co-, and Ni-based catalysts was estimated from rNH_3_w for the powder catalyst and the apparent density of the catalyst pellets. For example, in the case of Ru/Ba-Ca(NH_2_)_2_, the catalyst powder after ammonia synthesis reaction to measure rNH_3_w was pressed (300 MPa) into pellets in an Ar-filled glove box without exposure of the catalyst to the atmosphere. rNH_3_v for the catalyst was calculated from the apparent density of the resultant pellet and rNH_3_w for the catalyst powder. However, this value overestimates the ammonia synthesis rate/catalyst pellet volume; rNH_3_w for the catalyst particles in the powder catalyst is somewhat higher than that in pellets because of the diffusion limitation, and the estimated rNH_3_v for a catalyst is therefore expected to be somewhat higher than the ammonia synthesis rate of the actual catalyst pellets. As a result, the true rNH_3_v for Ru-, Co-, and Ni-containing catalysts is less than the corresponding rNH_3_v shown in Table S1.

^e^ Ammonia formation rate estimated based on rNH_3_w at a WHSV of 72000 mL g^–1^ h^–1^.

**Table S2.** Reaction orders (α, β, and γ for N_2_, H_2_ and NH_3_, respectively) over each iron catalyst.

| Catalyst | α | β | γ |
| --- | --- | --- | --- |
| p-Fe | 0.9 | 2.3 | -0.8 |
| AlH/Fe | 1.7 | 3.4 | -2.5 |
| AlH-K^+^/Fe | 1.5 | 7.6 | -3.9 |
| Promoted-Fe | 2.9 | 7.4 | -7.2 |


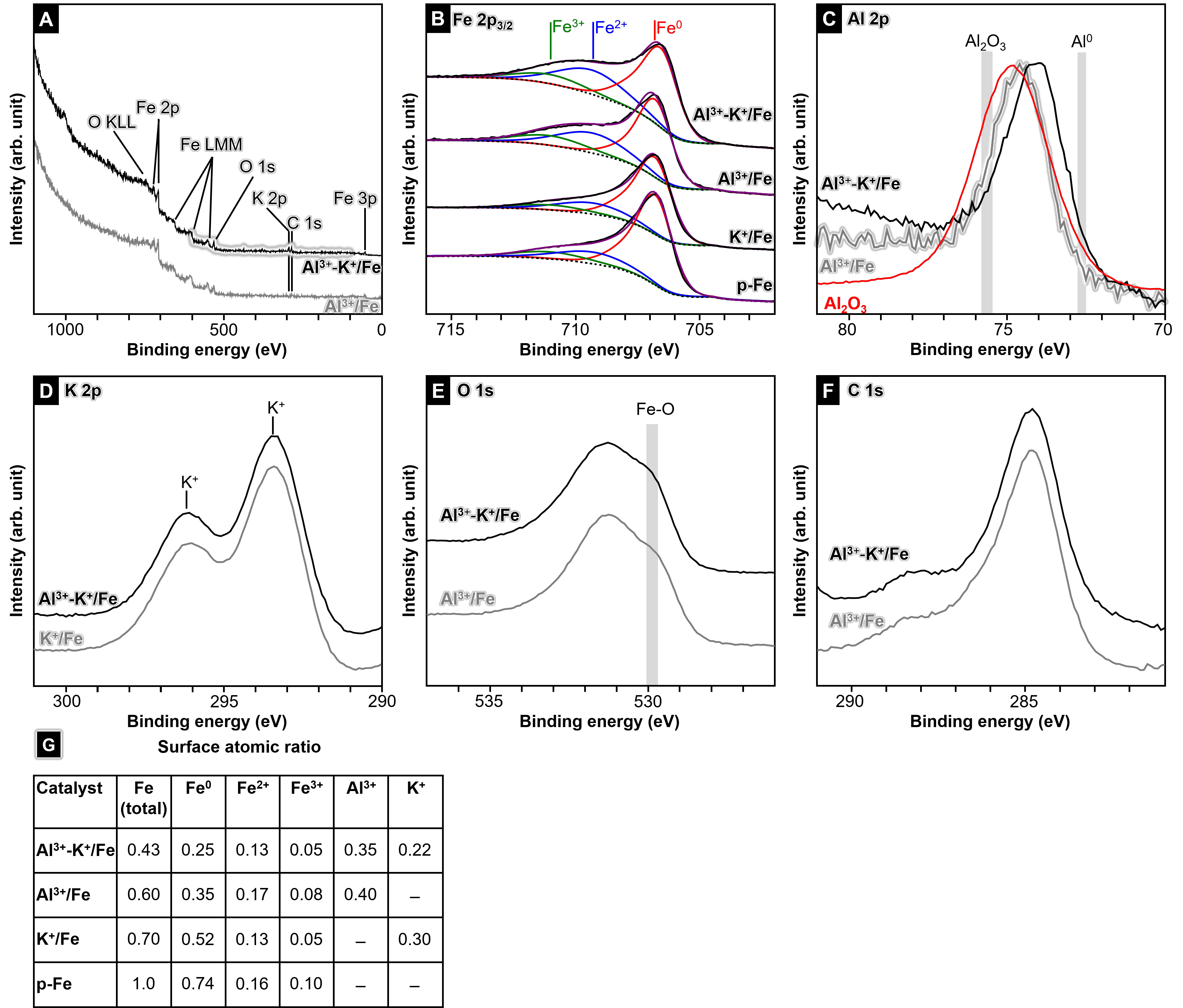


**Figure S1.** XPS results for the prepared iron catalysts after ammonia synthesis reached a steady-state (400 °C, 0.9 MPa). A) Survey spectra for Al^3+^-K^+^/Fe and Al^3+^/Fe, B) Fe 2p (Al^3+^-K^+^/Fe, Al^3+^/Fe, K^+^/Fe and p-Fe),^[6]^ C) Al 2p (Al^3+^-K^+^/Fe, Al^3+^/Fe and γ-Al_2_O_3_), D) K 2p (Al^3+^-K^+^/Fe and Al^3+^/Fe)^[7]^, E) O 1s (Al^3+^-K^+^/Fe and Al^3+^/Fe), F) C 1s (Al^3+^-K^+^/Fe and Al^3+^/Fe), G) Surface atomic ratio.


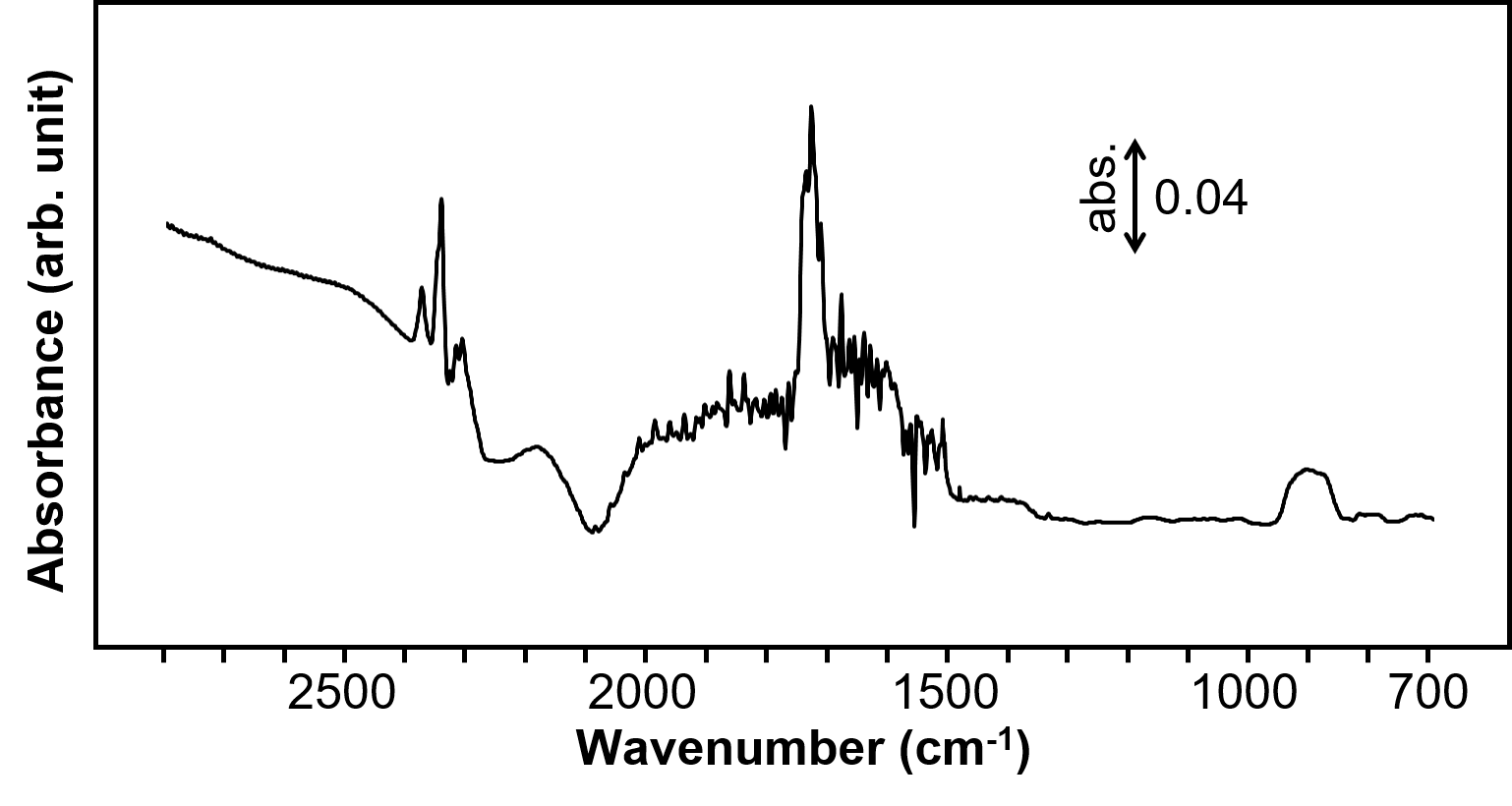


**Figure S2.** A FT-IR spectrum for Al^3+^-K^+^/Fe after ammonia synthesis. α-Fe_2_O_3_ precursor　(for Al^3+^-K^+^/Fe) double-layered self-supported disk (α-Fe_2_O_3_/SiO_2_) in the FT-IR cell was heated for 24 h at 400 °C in a flow (60 mL min^–1^) of N_2_-H_2_ (N_2_:H_2_ = 1:3) under atmospheric pressure, which resulted in the formation of AlH-K^+^/Fe, followed by cooling to 25 °C in a flow of Ar. The spectrum was obtained after the FT-IR cell was evacuated under vacuum (<1.3 Pa).


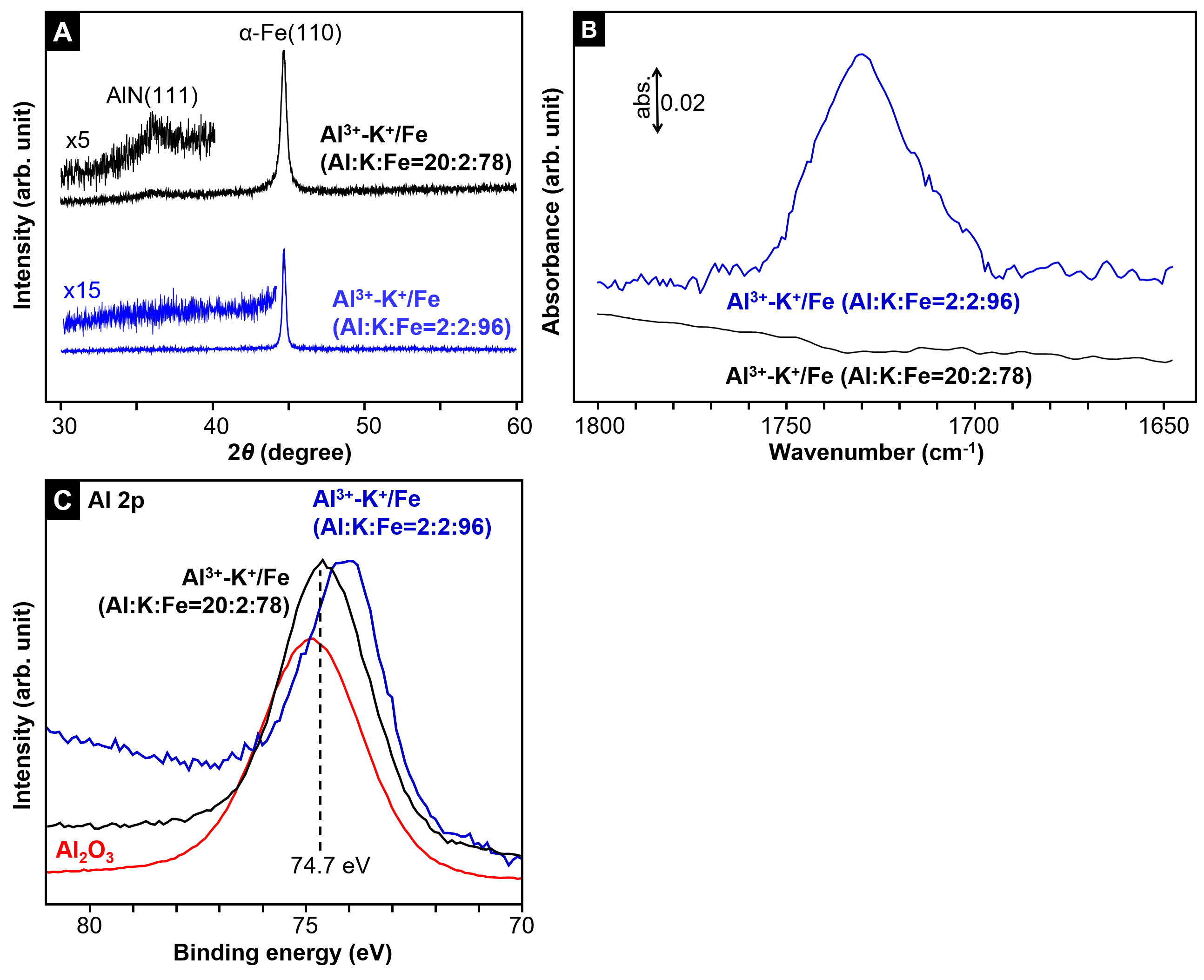


**Figure S3.** A) XRD patterns for the prepared iron-based materials (Al^3+^-K^+^/Fe (atomic ratio of Al/K/Fe=2/2/96 and 20/2/78)). B) FT-IR spectra for Al^3+^-K^+^/Fe after ammonia synthesis. α-Fe_2_O_3_ precursor (for Al^3+^-K^+^/Fe) double-layered self-supported disk (α-Fe_2_O_3_/SiO_2_) in the FT-IR cell was heated for 24 h at 400 °C in a flow (60 mL min^–1^) of N_2_-H_2_ (N_2_:H_2_ = 1:3) under atmospheric pressure, which resulted in the formation of AlH-K^+^/Fe, followed by cooling to 25 °C in a flow of Ar. The spectrum was obtained after the FT-IR cell was evacuated under vacuum (<1.3 Pa). C) XPS Al 2p spectra for Al^3+^-K^+^/Fe (atomic ratio of Al/K/Fe=2/2/96 and 20/2/78) after ammonia synthesis reached a steady-state (400 °C, 0.9 MPa). The γ-Al_2_O_3_ Al 2p spectrum measured by a spectrometer used in this study is shown in this figure for comparison.


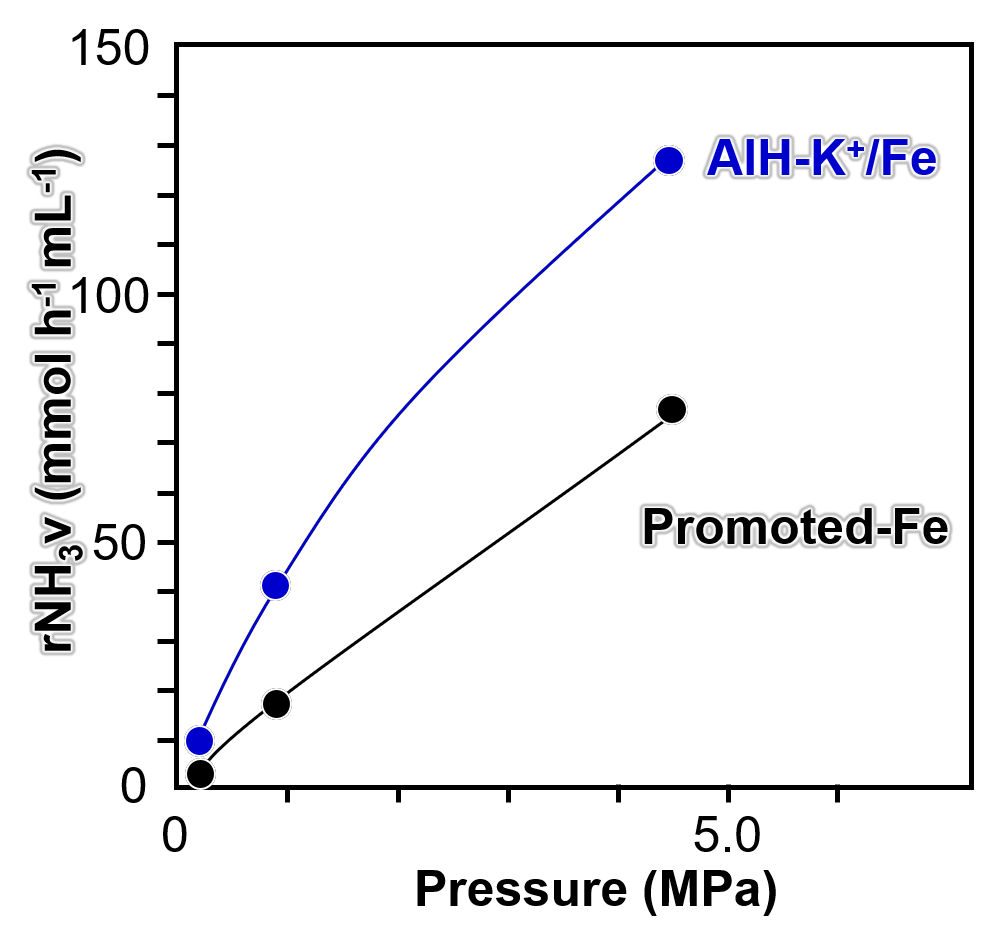


**Figure S4.** Correlation of rNH_3_v with pressure over AlH-K^+^/Fe and Promoted-Fe (400 ℃).


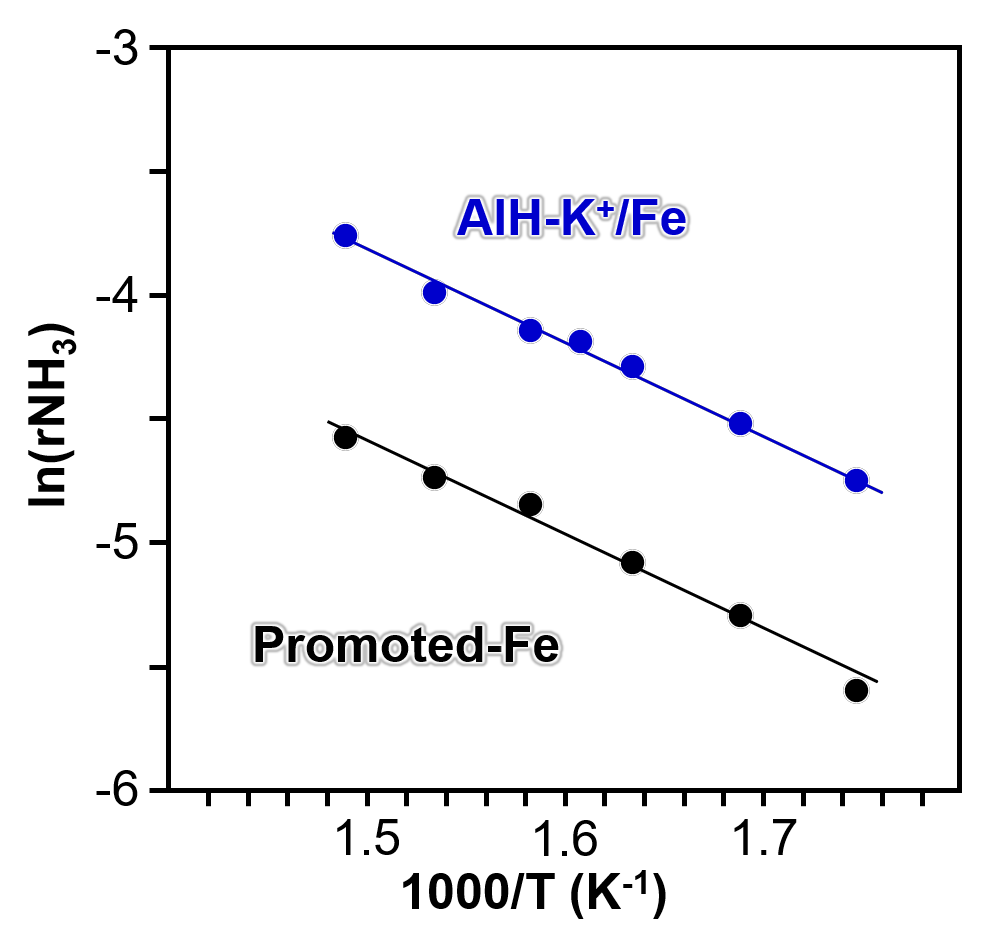


**Figure S5.** Arrhenius plots for AlH-K^+^/Fe and Promoted-Fe (0.9 MPa). There was no significant difference in the apparent activation energy, whereas there was a difference the ln(ammonia synthesis rate)-intercept (pre-exponential factor in the Arrhenius equation) between the two Arrhenius plots. This implies that AlH-K^+^/Fe has more active sites than Promoted-Fe.


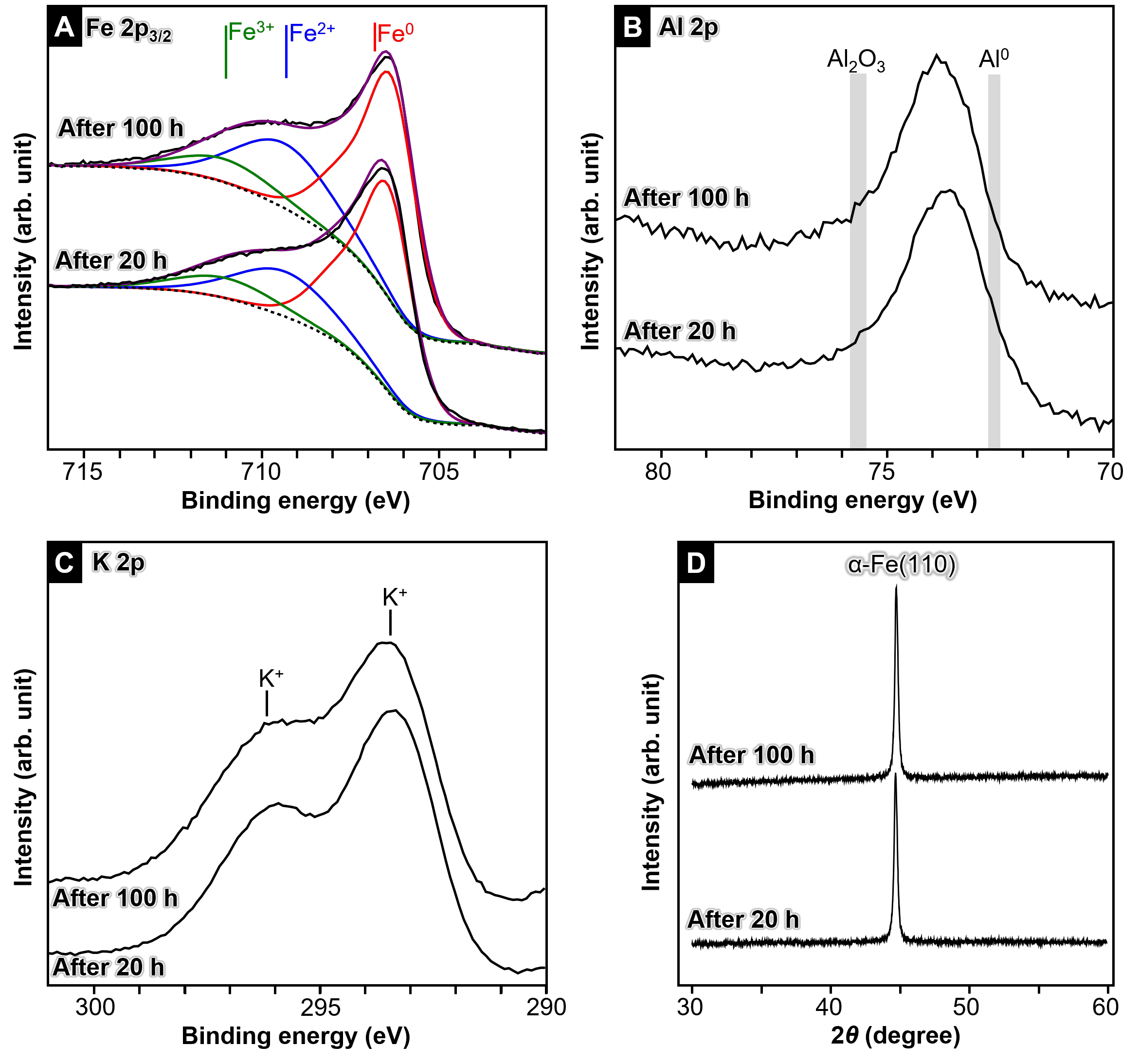


**Figure S6.** A-C) XPS spectra and D) XRD profiles for AlH-K^+^/Fe after the ammonia synthesis reaction for 20 and 100 h (400 °C, 0.9 MPa).


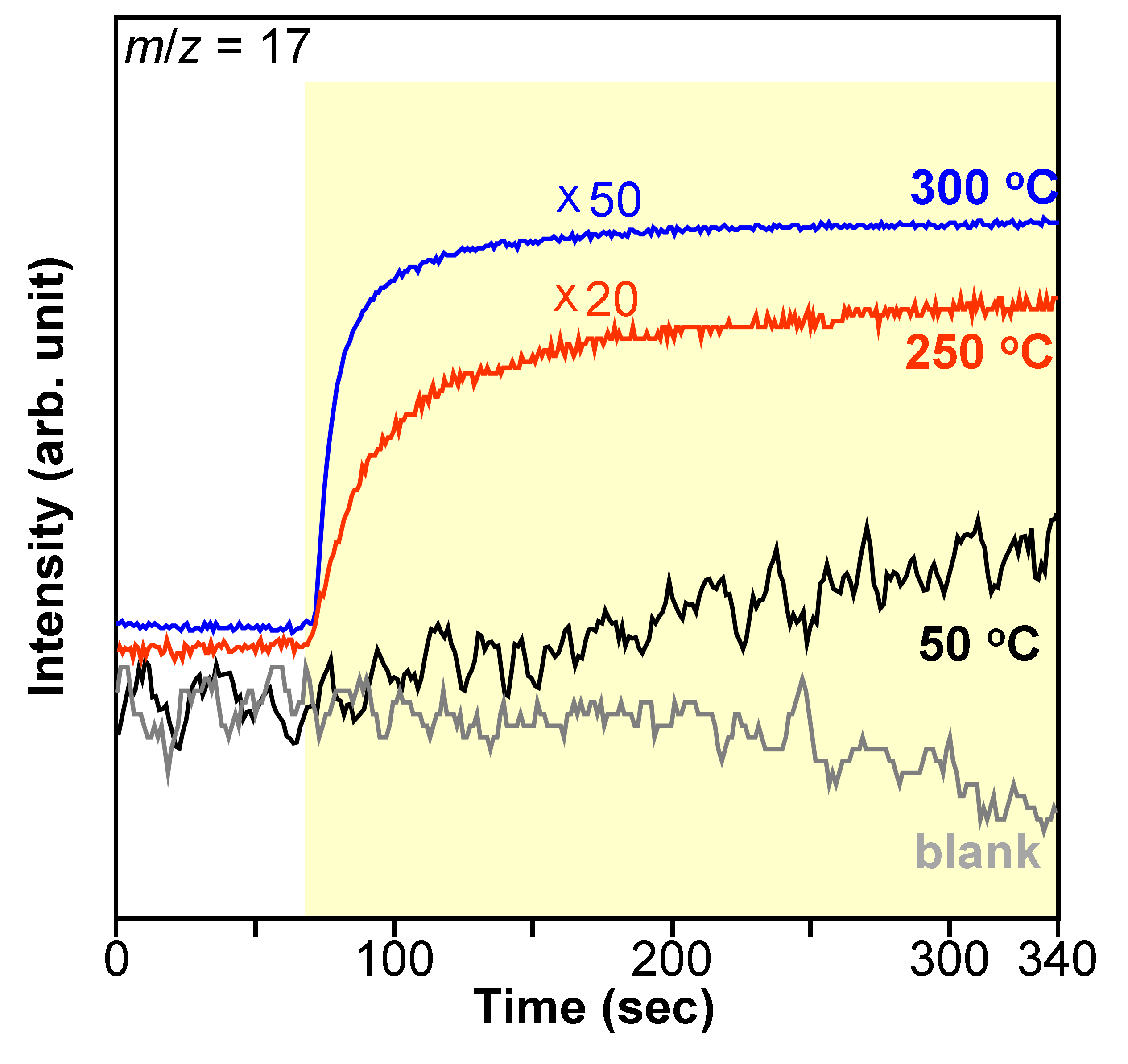


**Figure S7.** Time courses of m/z=17 for AlH-K^+^/Fe under ammonia synthesis conditions. AlH-K^+^/Fe in a steady state at 400 ℃ was cooled to the indicated temperature in a flow of Ar. After the catalyst was held each temperature, N_2_-H_2_ was introduced to the reactor at 0 second in the figure.


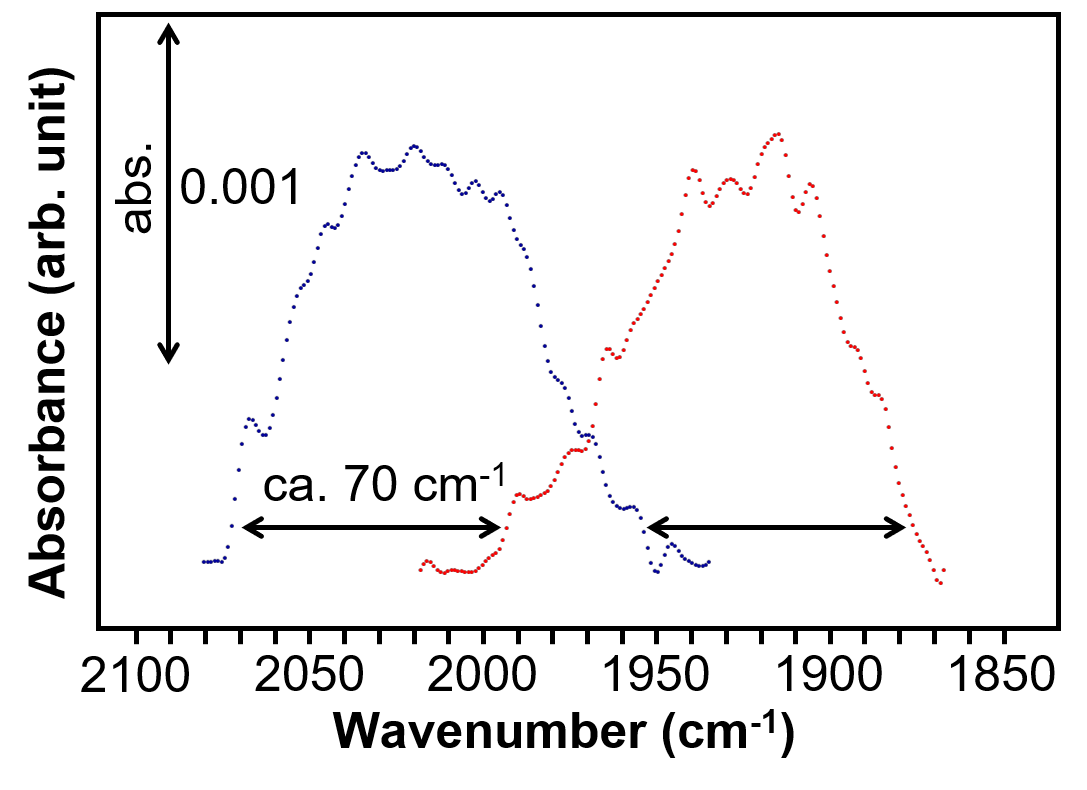


**Figure S8.** FT-IR spectra for ^14^N_2_ (blue)- and ^15^N_2_ (red)-adsorbed AlH-K^+^/Fe at 25 °C.

The broad band at 1950-2070 cm^-1^ for the ^14^N_2_-adsorbed catalyst appeared at 1870-2000 cm^-1^ in the spectrum for the ^15^N_2_-adsorbed catalyst. The red-shift of 70-80 cm^-1^ was consistent with the isotope effect between ^14^N_2_ and ^15^N_2_ (k’=k (14/15)^1/2^, where k’ and k are the wavenumbers for ^15^N_2_ and ^14^N_2_ stretching vibrations, respectively). The broad band at 1950-2070 cm^-1^ could thus be assigned to the N_2_ stretching vibration of N_2_ adsorbed on the iron catalyst.

**References**

1. M. Hattori, N. Okuyama, H. Kurosawa, M. Hara, *J. Am. Chem. Soc.* **2023**, *145*, 7888.
2. M. Kitano, Y. Inoue, M. Sasase, K. Kishida, Y. Kobayashi, K. Nishiyama, T. Tada, S. Kawamura, T. Yokoyama, M. Hara, H. Hosono, *Angew. Chem.* *Int.* *Ed.* **2018,** *130*, 2678.
3. K. Sato, S. Miyahara, K. Tsujimaru, Y. Wada, T. Toriyama, T. Yamamoto, S. Matsumura, K. Inazu, H. Mohri, T. Iwase, T. Taketsugu, K. Nagaoka, *ACS Catal.* **2021**, *11*, 13050.
4. T.-N. Ye, S.-W. Park, Y. Lu, J. Li, M. Kitano, T. Tada, H. Hosono, *Nature* **2020**, *583*, 391.
5. T.-N. Ye, S.-W. Park, Y. Lu, J. Li, M. Sasase, M. Kitano, H. Hosono, *J. Am. Chem. Soc.* **2020**, *142*, 14374.
6. N. S. Mcintyre, D. G. Zetaruk, *Anal. Chem.* **1977**, *49*, 1521.
7. R. Sawyer, H. W. Nesbitt, R. A. Secco, *J. Non-Cryst. Solids* **2012**, *358*, 290.
